# The Revision and Application of Aurora in China: Based on Successful Intelligence

**DOI:** 10.3390/jintelligence10040120

**Published:** 2022-12-06

**Authors:** Li Cheng, Jinglu Yan, Xiaochen Ma, Xiaoyu Chen, Zhengkui Liu

**Affiliations:** 1 Faculty of Education, Beijing Normal University, Beijing 100875, China; 2 Developmental and Educational Research Center for Children’s Creativity, Faculty of Education, Beijing Normal University, Beijing 100875, China; 3 Institute of Psychology, Chinese Academy of Sciences, Beijing 100101, China; 4 Department of Psychology, University of Chinese Academy of Sciences, Beijing 100101, China; 5 Institute for Social Science Research, The University of Queensland, Brisbane, QLD 4068, Australia; 6 Shenzhen Nanshan Longyuan School, Shenzhen 518052, China

**Keywords:** successful intelligence, Aurora Battery, analytical ability, creative ability, practical ability

## Abstract

Aurora Battery is a corresponding test of successful intelligence. This study aims to examine the factorial structure of the Chinese version of Aurora Battery and to investigate its internal consistency and validity, as well as to discover the developmental features of Chinese students. A total number of 2007 students were recruited from 13 schools across eastern, central, and western China, ranging from 4th to 8th grade (mean age = 12.29 years) and among them, 43.9% are girls. Confirmatory factor analysis (CFA) was used to examine the factorial structure. Among the tested models, a second-order factor model, in which the three ability factors serve as indicators of a general factor, provided an acceptable model fit to the data. Moreover, measurement invariance across gender and grades were supported, which suggests the mean scores of analytical, creative, and practical abilities are comparable in this research. The criterion-related validity analysis suggests that the battery and its three subscales have good criterion validity. The scale reliability analysis shows that the Cronbach’s alpha and the McDonald’s omega value of the whole test were .84 and .87, respectively, indicating the scale’s internal reliability is good. For ability differences among grades, students’ analytical and practical abilities increase across all grades, while creativity presents an upward trend from grade 4 to 6, followed by a downward trend from grade 6 to 7, and an increase from grade 7 to 8. Female students outperform male students on both analytical and creative ability, while with no obvious difference on practical abilities.

## 1. Introduction

One of the greatest challenges in the field of education is how to identify students’ cognitive abilities. In previous research, standardized academic achievement tests and traditional IQ tests are the most frequently used tools to assess children’s cognitive abilities (Gubbels et al. 2016; Mandelman et al. 2016), such as The Wechsler Intelligence Scale for Children (WISC) (Canivez 2014), the Scholastic Assessment Test (SAT), or American College Testing (ACT) (Kuncel et al. 2001; Noble and Sawyer 2002; Shaw et al. 2011; Stilwell et al. 2011; Talento-Miller and Rudner 2005). All of these traditional IQ tests and standardized academic achievement tests have been proven to have good reliability and validity, and can be helpful in identifying students’ strengths and weaknesses in learning. However, experts argue that these tests are still a bit narrow to some extent, as they are more of “general ability”, which always emphasize memory-based and analytical skills (Sternberg 2015). Consequently, the currently used academic achievements or traditional IQ tests are less than comprehensive, since success in real life is dependent on a wider range of abilities than what academic achievements tests or traditional IQ tests measure. Those traditional tests may result in unrecognized and un-nurtured abilities, and as a consequence, may have long-term devastating effects (Mandelman 2013). For example, researchers discovered that in the USA, students with other abilities which were not recognized in traditional IQ tests or academic tests, are usually excluded from the gifted programs, and this situation is more severe among students from minority groups and low-SES backgrounds (Gubbels et al. 2016; Rowe 2005; Sternberg et al. 2005). Specifically, spatial ability, an important determinant for scientific breakthroughs, is often neglected by giftedness search procedures, since the procedures focus on evaluating the individual’s mathematical and verbal ability, which lead to an situation that the vast majority of spatially gifted students are refused by giftedness search programs (Kell and Lubinski 2013).

Under this circumstance, academic institutions have delineated students’ cognitive abilities more broadly. Allan classifies students’ cognitive abilities into three types, specifically, subject-based abilities, personal transferable abilities (comprising acting independently, collaborating with others, using information, effectively communicating), and generic abilities (including thinking critically, analyzing, synthesizing ideas and information) (Allan 1996; Phillips et al. 2010). Sternberg uses a similar structure to evaluate children’s cognitive abilities, which is the theory of successful intelligence, and he also compiles some tests for this theory (Chart et al. 2008). This theory not only provides us with a broader horizon in understanding the student’s ability, but also redefines and addresses the nature of what is crucial for individuals’ long-term success (Mandelman 2013).

Successful intelligence is defined as a person’s ability to achieve their goals in real life, within his or her immediate environment (Sternberg et al. 2010). The kernel behind successful intelligence theory is that the success of real life is driven by three integrated key features: analytical ability, which is more of a traditional intelligence and academic achievement, refers to the ability primarily measured by traditional academic tests, by which children are enabled to analyze, evaluate, judge, compare and contrast (Mandelman et al. 2010); creativity, which reflects the individual’s capacity to generate original and effective ideas, and can be conducive for raising good questions and forming excellent ideas from a unique and innovative perspective (Runco and Jaeger 2012). Though sometimes criteria such as the creative product should be surprising (Simonton 2012), esthetic value and authenticity (Kharkhurin 2014) were added to evaluate the quality of creativity, originality and usefulness are still two essential and fundamental criteria (Karwowski et al. 2016; Runco and Jaeger 2012); and practical ability emphasizes an individual’s ability to carry out good ideas effectively (Sternberg 2011). The analytical ability, during processes of problem-solving and decision-making, can help people assess opportunities and make favorable choices even in complex situations. As for creativity, more recently, a lot of researchers tended to hold the view that it is a subcomponent of intelligence (Karwowski et al. 2016), and those two constructs might be more similar than previously regarded (Gerwig et al. 2021; Silvia 2015). For example, in a meta-analysis, researchers discovered that the correlation between intelligence and divergent thinking, which is an indicator of creativity, can reach up to .31 to .37, after considering the influence of some moderators). Moreover, meta-analyses also demonstrated that academic achievement, which reflected from analytical abilities, has a significantly positive relationship with creativity (Karwowski 2021). Though sometimes intellectual gifted individuals do not always outperform in creativity tasks, above-average intelligence is often considered to be beneficial for improving the quality of creativity (Karwowski et al. 2021). Vice versa, creativity can also better help individuals apply analytical skills, which are more frequently reflected through traditional IQ tests and academic achievement tests (Karwowski 2021). Practical ability, in this context, is conceptualized as a sort of tacit knowledge obtained through experiences in everyday life, which undergoes continuous development (Sternberg 2003). Practical abilities can help individuals better adapt to their environment, and deal with problems of daily life (Aljughaiman and Ayoub 2013), and through practical ability, the individual’s analytical ability and creativity can be better applied and transferred and they can achieve their goals (Aljughaiman and Ayoub 2012). Overall, analytical, creative, and practical ability are three distinct but interrelated components of an individual’s cognitive abilities, and in order to achieve success, individuals consolidate their strengths and remedy their weaknesses through the integral use of analytical, creative, and practical abilities (Sternberg 2005; Sternberg and Grigorenko 2007; Sternberg et al. 2009). By doing this, successfully intelligent individuals can better adapt to environments, as well as actualize their full potential through the balanced use of their analytical, creative, and practical abilities (Mandelman et al. 2016). 

Though the theory of successful intelligence gave us some insights about individuals’ cognitive abilities, some researchers criticized that the gist of Sternberg’s theory was not something new. Before Sternberg, many scholars had put forward that intelligence should not just be a single factor. For example, some scholars proposed that intelligence was constituted of several primary mental abilities instead of one general factor, such as word fluency, or inducive reasoning (Gubbels 2016); moreover, Cattell classified intelligence into fluid and crystalized intelligence; the former one is similar to Sternberg’s analytical ability, while the latter is identical to the practical ability (Hunt 2008). In addition, even for the component of creativity, Torrance and colleagues, as well as Renzulli, all had realized that creativity should be another independent factor of intelligence (Plucker 1999; Renzulli and Reis 2018). Furthermore, in empirical studies, Sternberg claimed that assessments based on successful intelligence has significant correlations with the Concept Mastery Test, the Watson–Glaser Critical Thinking Appraisal, the Cattell Culture-Fair test of g, and a test of creative insight constructed by him and his colleagues (Sternberg et al. 1996), and can both predict academic performance accurately and individuals’ other characteristics, which are pertinent to success in everyday life. For instance, the Kaleidoscope Project (Sternberg et al. 2012), which used the successful intelligence test as an optional supplementary test for students at Tufts University, found that students who were measured by the Kaleidoscope Project performed academically as well as their peers who were tested by traditional measures. However, students who achieve higher scores in this project tended to participate in more extracurricular and leadership related activities, which are proposed to be a positive index for future competence (Sternberg 2010); meanwhile at workplace settings, Sternberg and colleagues obtained significantly positive correlations between tacit knowledge, the kernel of practical intelligence, and employees’ merit-based salary increase and work performance rating (Sternberg 2000). However, Brody and Gottfredson pointed out that, if statistical criteria for data summarization were applied to Sternberg’s studies, the correlations were lowered, and there is little advance beyond the g-factor of intelligence (Brody 2003; Gottfredson 2003). Nevertheless, we should notice that the theory of successful intelligence integrated those separately sold ideas of intelligence, and carried out it in educational settings, this is a nontrivial practice, and it made the criteria for education changed and become more multi-facet. From this perspective, it is worthy to contribute efforts and further investigate this theory. Apart from this, in China, educational reform is constantly deepening, emphasizing that the fundamental task of education is to foster virtue through education. Guided by this goal, the Chinese government promulgated the “*Overall Plan for Deepening the Reform of Education Evaluation in the New Era*” in 2020, clearly proposing altering the evaluation mechanism based solely on subject scores and paying more attention to the improvement of students’ comprehensive quality, practical ability, and core literacy. This is basically consistent with the core abilities emphasized by successful intelligence. Therefore, adopting the theory of successful intelligence to evaluate students’ cognitive abilities is in accordance with the requirements of China’s educational reform, and it can provide an option for children to have a more comprehensive evaluation of their strengths and weaknesses. The Aurora Battery, a newly designed test for successful intelligence, had made great changes in comparison with previous tests: the modalities were more diverse than before, and the three abilities were evaluated across various domains. Therefore, using this new approach to evaluate Chinese students’ successful intelligence is worthwhile. 

Thus, the purpose of this article was to translate and revise the Chinese version of the Aurora Battery, examining the factorial structure of this assessment and investigating its internal consistency and validity. The Chinese version of the Aurora Battery was then further used to measure the successful intelligence level of Chinese students by analyzing the gender and grade differences of the three abilities. A sample of 2007 students from 4th grade to 8th grade was comprised in this study. This broad age spectrum and the large sample would provide a clearer look at the developmental trend of successful intelligence among Chinese children and adolescents (Lau and Cheung 2010).

## 2. Materials and Methods

### 2.1. Participants

The sample for this study comprised 2007 students (46.9% female) in the age of 9.05 to 15.86 years (*M* = 12.29, *SD* = 1.41). Stratified random sampling was used to recruit participants from 4th to 8th grade in Eastern, Central, and Western China. Table 1 shows the basic demographics of the sample. 

In order to assess the criterion-related validity, another 443 students from 4th, 5th, 7th, and 8th grade were recruited to finish Aurora Battery, Test of Nonverbal Intelligence-Second Edition (TONY-2) and Evaluation of Potential for Creativity (EPoC). Among them, 51.5% are female (N = 228), aging from 8.87 to 15.03. Specifically, 24.6% from 4th grade (N = 109), 30.7% from 5th grade (N = 136), 22.3% from 7th grade (N = 99), and 22.3% from 8th grade (N = 99).

### 2.2. Measurement

The Chinese Version of Aurora battery. The Aurora Battery is an augmented assessment that measures abilities in the areas of analytical, practical, and creative thinking. The subtests were designed to assess these abilities across stimulus domains (verbal, numerical, and images) and item formats (multiple choice, open ended) such that a balanced range of opportunities could be offered for children to demonstrate various abilities within and across domains (Chart et al. 2008). Two psychology graduate students translated the Aurora Battery into Chinese and then a third PhD student did the back translation. For those back-translated items which were different from the original version, we invited a professor of psychology to discuss with the translators and confirmed the final Chinese expression of these items. Because the homophones subtest items were related to language pronunciation, the Chinese version items needed to be redesigned. We invited four experienced literature teachers to compile 40 homophones in Chinese for the homophones subtest. Then, we invited two students from each grade, in grades 4 to 8, to conduct structured interviews to collect their opinions on the difficulty and cultural appropriateness of the 40 homophone items. According to the interview results, we finally chose 20 items from this list to use. 

In all, 388 students (aged from 10–14, 52.3% female) were invited to participate in a pilot study that sought to determine the understandability and difficulty of the translated items. As a result of this pilot study, the Silly Headlines and Shapes (Abstract Tangrams) subtests were deleted due to cultural difference and difficulty level. For instance, Silly Headlines were considered as a kind of American slang, which made it hard for Chinese students to understand the humor implied in it. While Shapes (Abstract Tangrams) were too easy for Chinese students to answer so that a ceiling effect existed and could not evaluate students’ abilities accurately. The final version of the translated Chinese Aurora Battery is shown in Table 2. The battery consisted of 15 subtests that comprised 120 items.

Test of Nonverbal Intelligence-Second Edition (TONI-2). TONI is a language and culture free intelligence test built by Brown et al. (Brown et al. 1982) and was revised in 1990 (Brown et al. 1990) as TONI-2, which is used to test nonverbal abstract/figure problem solving abilities for 5 years to 85 years old in eight areas including shape, position, direction, rotation, contiguity, shading, size and movement. Zhang and Cha (2003) revised Chinese mainland TONI-2 version according to the Form of Taiwan Version (Wu et al. 1996) and rebuilt the norm in mainland China (Zhang and Cha 2003), and then updated the norm in 2011. The Chinese version of TONI-2 includes 63 items, and its Cronbach’s alpha was .89, which was acceptable to good consistency.

Evaluation Potential of Creativity (EPoC). Evaluation Potential of Creativity (EPoC) was constructed by Lubart et al. (Barbot et al. 2011), and it consists of a total of eight tasks, four of which are “Convergent-Integrative (CI)” and the other four are “Divergent- Exploratory (DE)” tasks, which applied in two content-domains: Verbal-literary (V) and Graphic (G). We measured the creativity of participants in the following four aspects: (1) divergent verbal (DV), the participants were given the beginning of a story, and they were required to imagine and write as many possible endings of the story as they can; (2) divergent graphics (DG), the participants were given an abstract or concrete graphic, and they were required to paint as many pictures as they can on the basis of the given figure; (3) integrated verbal (IV), the participants were given three story elements, and they were required to write an original story according to the elements; (4) integrated graphics (IG), the participants were given eight abstract or concrete graphics, and they are required to select at least four of them to create a novel picture. The divergent tasks were rated for fluency, while the convergent tasks were scored for originality, the raw scores of each task were standardized into z-scores. For this study, the inter-rater agreements on IV and IG were all above .9, with the Cronbach’s alpha values of .97 and .95, respectively.

### 2.3. Procedure

Before conducting this research, the ethical approval was received from the University of Queensland (approval number: 2020000934). We provided the information letter and consent form to both children and their guardians separately, to ensure that all the participants and their guardians understood the purpose and procedures of this study. Based on the voluntary principle, consent from students and their guardians were obtained. Graduate students in pedagogy and psychology who received comprehensive training, instructions, and experimental record lists carried out data collection at the participating schools. Investigators recorded information on the number of participating students, grade, gender, the order of subtest administration, time students used to finish each test, as well as the questions students asked while doing the tests. Head teachers assisted in emphasizing the importance of the test. For primary school students (4th to 6th grade), head teachers used the same instructions and answered students’ questions under the guidance of investigators while middle school (7th to 8th grade) students finished all the tests themselves. The battery was split into three parts (i.e., analytical tests, creativity tests, and practical tests) and the order of the tests was counterbalanced across classes, grades, and schools. Primary school students were asked to finish it within one hour per part whereas middle school students had 45 min each for analytical and practical tests, and one hour for creativity test. 

### 2.4. Data Analysis

Each open-ended subtest (Metaphors, Book Covers, Conversations, Multiple Uses, Number Talk) was scored by two raters who had been trained to reach a satisfactory agreement more than .8 in their scoring using a standardized rubric. Both raters scored 10% of the same students for each subtest. To calibrate the scores of students rated by only one rater, the mean and standard deviation (*SD*s) for the overlapping scores between raters were computed (Ferrando et al. 2016). Next, the mean was subtracted from the raw score and then divided by the standard deviation for the total sample to calculate z-scores for each participant for all the items per subtest (Mourgues et al. 2016). Then, researchers multiplied these *z*-scores by 10 and added 50 to obtain *T*-scores for further analysis. As for other 10 subtests (Floating Boats, Homophone, Story Problems, Number Cards, Figurative Language, Paper Cutting, Toy Shadows, Decisions, Maps, and Money Exchange), raw scores of each subtest were standardized as z-scores, and then the z-scores were transformed into *T*-scores, with the mean value of 50 and SD of 10. 

A set of confirmatory factor analysis (CFA) was conducted to determine the factor structure of the Aurora Battery. Specifically, three models were tested and compared for their respective fit to the data. Model 1 specified one second-order factor of three domains. Model 2 estimated one second-order factor across the three abilities. Model 3 was a correlated trait-correlated method minus one model (CTC(M-1)), composed of three ability factors, which are analytical, practical, and creative ability, and with one domain factor less than domains considered. In Model 3, the images domain was chosen as the comparison standard. 

After identifying the best-fitting model of the Aurora Battery, we evaluated measurement invariance (MI) across gender and grades. All models were estimated by Mplus 7.4 (Muthén and Muthen 2017), using maximum likelihood (ML) estimator. The estimates were obtained through the expectation-maximization (EM) algorithm. And for the missing value, the point estimate was filled in on the basis of the ML estimates of the means and covariances (Charalambous and Logothetis 2000). In order to adopt the EM algorithm, it was hypothesized that the data were multivariate normal and that the missingness was at random. Although simulations indicate that the EM algorithm is quite robust to violations of the multivariate normality assumption, we still checked the skewness and kurtosis of the score distribution, as the skew and kurtosis ranges from −1.5 to 1.5, it suggested that the distribution of this data can be regarded as close to normal (George and Mallery 2018).

Multiple fit indices were used to evaluate the goodness-of-fit of each model: Comparative Fit Index (CFI) and Tucker-Lewis index (TLI) with value ≥ .9 indicating an acceptable fit, and with value ≥ .95 suggesting a good fit; and Root Mean Square Error of Approximation (RMSEA) with values ≤ .06 suggesting an accepted fit of the model to the data, and a Standardized Root Mean Square Residual (SRMR) with values less than ≤.80 indicating an appropriate fit (Bentler 1995; Byrne 2006; Xie et al. 2022). Moreover, changes in CFI and RMSEA (Δ) were also employed to compare the nested models, with the values of ΔCFI ≤ .01, and ΔRMSEA ≤ .015 indicating the difference is not significant (Chen 2007; Cheung and Rensvold 2002). The chi-square test of the model fit was also reported, however, this results relied heavily on sample size (Cheung and Rensvold 2002). 

Correlations between the Aurora Battery and its subscales with TONY-2, EPoC and academic performance at school were also analyzed separately to assess the criterion-related validity.

As for reliability of the scale, omega (ω), omega subscale (ωS) and Cronbach’s alpha were computed to calculate the internal consistency of the Aurora Battery and its subscale.

A descriptive analysis of the Aurora Battery scores on Chinese students was conducted. ANOVA and *t*-test were adopted to probe whether there were gender and grade differences on those three abilities.

## 3. Results

### 3.1. The Factorial Structure of the Chinese Aurora Battery

**Figure 1:**
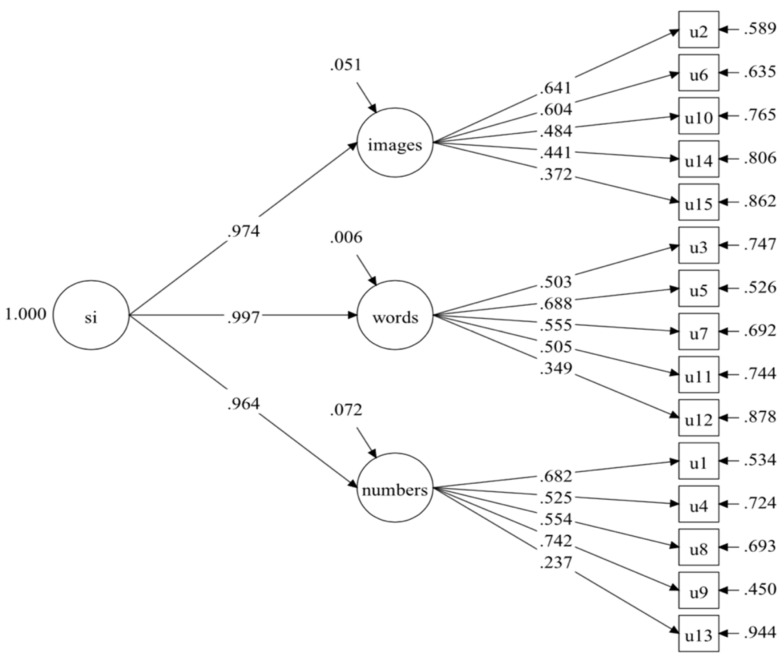
Model 1: one general factor of three domains. Notes. u1 = Story Problems, u2 = Floating Boats, u3 = Metaphors, u4 = Number Cards (Letter Math), u5 = Words That Sound the Same (Homophones), u6 = Paper Cutting, u7 = Decisions, u8 = Maps, u9 = Exchange, u10 = Toy Shadows, u11 = Interesting (Figurative) Language, u12 = Conversations, u13 = Number Talk, u14 = Multiple Uses, u15 = Book Covers.

**Figure 2:**
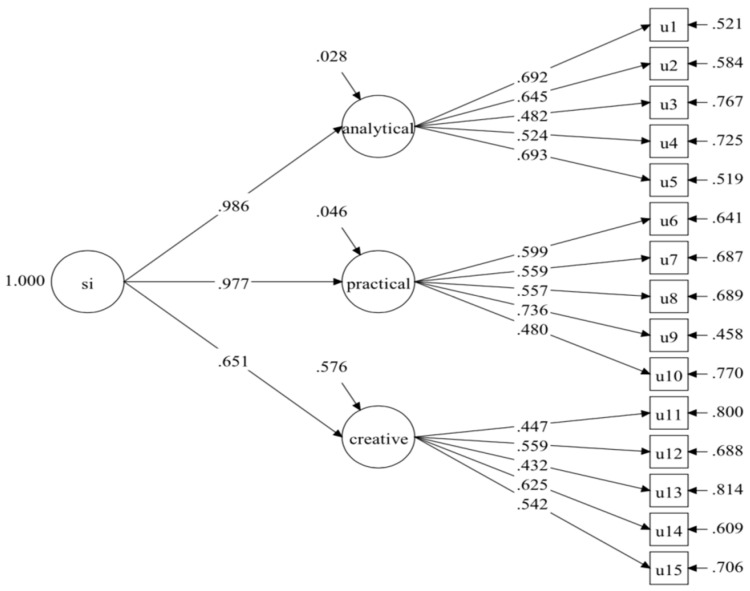
Model 2: one general factor of three abilities. Notes. u1 = Story Problems, u2 = Floating Boats, u3 = Metaphors, u4 = Number Cards (Letter Math), u5 = Words That Sound the Same (Homophones), u6 = Paper Cutting, u7 = Decisions, u8 = Maps, u9 = Exchange, u10 = Toy Shadows, u11 = Interesting (Figurative) Language, u12 = Conversations, u13 = Number Talk, u14 = Multiple Uses, u15 = Book Covers.

**Figure 3:**
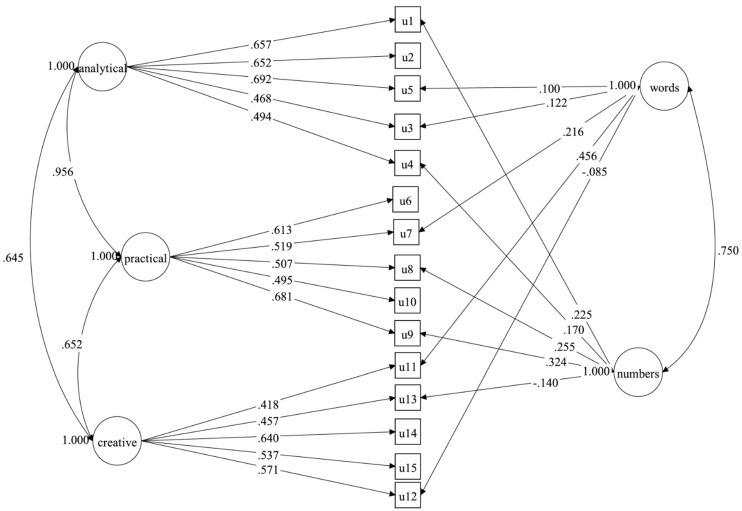
Model 3: CT-C(M-1) model. Notes. u1 = Story Problems, u2 = Floating Boats, u3 = Metaphors, u4 = Number Cards (Letter Math), u5 = Words That Sound the Same (Homophones), u6 = Paper Cutting, u7 = Decisions, u8 = Maps, u9 = Exchange, u10 = Toy Shadows, u11 = Interesting (Figurative) Language, u12 = Conversations, u13 = Number Talk, u14 = Multiple Uses, u15 = Book Covers.

The inter-rater agreement of the five open-ended subtests between the raters were assessed by percentage of agreement between raters, ranged from .96 to .98 (Conversations), .97 to .99 (Metaphors), .96 to .97 (Number Talk), .80 to .96 (Book Covers), and .88 to .94 (Multiple Uses). Next, three different models were constructed to examine whether students’ performance was better explained by a general factor of intelligence through domain-specific or through ability-specific factors of intelligence, or whether it can both present ability and domain traits. Model 1 (see Figure 1) and Model 2 (see Figure 2) were two second-order models based on three domains and abilities, respectively. Model 3 (see Figure 3) was a CT-C(M-1) model that was composed of three ability factors, that is analytical, practical, and creative abilities, and two method factors (i.e., domain factors), which included the words, and numbers, while the images domain was set as a reference group. Table 3 shows the model fit indices of all tested models. Although model 3 showed the best fit indices among all models that were tested, some of the factor loadings on the words and numbers domain were rather low, suggesting that a model based on abilities or domains may be more appropriate to fitting the empirical data. Compared to the first two models, Model 2 obtained better fit indices, and compared to Model 1, the changes in CFI (|ΔCFI| = .041), TLI (|ΔTLI| = .049), and RMSEA (|ΔRMSEA| = .005) were significantly better, thus, Model 2 was considered as the final model and would be used for further analysis. Table 4 shows the decomposition of variance for Model 2.

Multigroup CFA was adopted to examine MI across grades and gender. Following the procedures put forward by previous research, seven levels of invariance were tested, and male group and grade 4 was set as the reference group. First, configural invariance of the first-order factors (M1) were tested, at this first level, invariance required the number and pattern of factors of the overall baseline model structure to be equal across grades and gender. Following was tests of first-order and second-order metric invariance, which are necessary predictions to examine the first- and second-order scalar invariance. For first-order metric invariance model (M2), all loadings of observed variables on first-order factors (e.g., analytical, practical, and creative ability) were constrained to be equal across grades and gender; whist for the second-order metric invariance model (M3), all second-order factor lodgings were additionally constrained to be equal. For the first-order scalar invariance model (M4), intercepts of observed variables were constrained to be equal; and for the second-order scalar invariance model (M5), intercepts of first-order factors were additionally constrained to be equal. Partial invariance was also examined when the full scalar invariance was not fully supported. After that, first-order residual invariance (M6) was tested, by constraining all errors of the observed variables equal across grades and gender. This type of invariances in measurement errors could clarify whether grade-related and gender-related differences on the observed variables were attributable to grade-related and gender-related differences on the corresponding latent variables. Finally, for the last level, the invariance of the disturbances of the first-order factors (M7), apart from all the previous constraints, disturbances of all first-order factors were set to be equal across grades and gender. If this level of invariance could be achieved, it suggests the disturbances of the lower order factors will be equivalent across the various grades and gender.

All fit indices were presented in Table 5 and Table 6. Among those tested models, most models met the requirements of changes in CFI (|ΔCFI| ≤ .01), and RMSEA (|ΔRMSEA| ≤ .015) were insignificant, one exception was the first-order scalar model across gender, the changes in CFI on this model exceeded the threshold of .01, which suggested a partial first-order scalar model (M4a) might be more appropriate. When the intercepts of subtest metaphor and toy shadow in female group were allowed to estimate freely, the changes in CFA of this model turned out to be insignificant in comparison with the second-order metric model (M3). Thus, MI across grades and gender were mostly supported, which indicates that the means of successful intelligence are comparable.

### 3.2. Criterion-Related Validity

Criterion validity assesses the accuracy a test measures the outcome it was designed to reflect, and it can be demonstrated by its correlation with the assessment that is already considered valid. When the correlation between the new test and the criterion is significant, it proves that the test has good validity (Rafilson and Sison 1996; Shih et al. 2022). 

Aurora, the academic performance test, TONI-2, and EPoC are all cognitive ability tests, of which TONI-2 and EPoC had been proved to have good reliability and validity and were widely used in various countries, regions, and populations. Therefore, the score of TONI-2 and EPoC could be used as criteria to verify whether the Aurora could test the cognitive ability of individuals. In accordance with the previous section, researchers computed correlations between Aurora Battery with different criteria separately (see Table 7).

Assessment of criterion validity shows that TONY-2 has significant positive correlations with the Aurora Battery score (r = .52, *p* < 0.001), analytical intelligence score (r = .48, *p* < 0.001), practical intelligence score (0.45, *p* < 0.001), and creative intelligence score (r = .42, *p* < 0.01). 

While for the association between the Aurora Battery and EPoC, correlation analysis indicates that it exists medium strong correlations, with the coefficient of .53 (*p* < 0.001) for the Aurora Battery total score, .48 (*p* < 0.001) for analytical intelligence score, .53 (*p* < 0.001) for creative intelligence score, and .33 (*p* < 0.001) for practical intelligence score. 

The academic test, almost the most important test in an educational setting, is always the focus of teachers and parents. As shown in Table 8, this 188 sample were chosen from the previous 2007 sample, who adopted identical test papers and scores obtained from the teachers, so that the accuracy of the academic scores could be guaranteed. Results showed that Chinese, math scores, as well as the total academic scores, were all positively associated with successful intelligence, with medium to strong correlations. The correlations between Chinese scores and success was .65 (*p* < 0.001) for the Aurora Battery total score, .62 (*p* < 0.001) for analytical intelligence score, .45 (*p* < 0.001) for creative intelligence score, and .53 (*p* < 0.001) for practical intelligence score. As for the math scores, the correlation was .57 (*p* < 0.001) for the Aurora Battery total score, .48 (*p* < 0.001) for analytical intelligence score, .40 (*p* < 0.001) for creative intelligence score, and .50 (*p* < 0.001) for practical intelligence score. With regard to total score of academic performance, the correlation with total Aurora score, analytical, creative, and practical intelligence score, were .72, .64, .50, and .61, respectively (*p* < 0.001). 

### 3.3. Internal Consistency

The Cronbach’s alpha of the total composite score (i.e., across items of all 15 subtests) was .84, and the Cronbach’s alpha of analytical ability, practical ability and creative ability were .75, .72, and .62. A lot of researchers criticized that Cronbach’s alpha is less reliable, so model-based omega coefficients are needed for further reliability investigation (Dunn et al. 2014). The omega for the total scale was .87, which indicates that 87% of the variance in item responding could be ascribed to the factors, and only 13% of the variance was because of errors. Omegas for each dimension were also calculated: omega for analytical subtest was .83, omega for creative subtest was .77, and omega for practical subtest was .82.

### 3.4. Descriptive Results

The mean score and standard deviation of various gender and grade among the three subscales are shown in Table 9.

According to Table 9, students in higher grade levels outperformed on both analytical and practical tests. With regard to creativity, students showed an upward trend on scores from grades 4 to 6, whereas with a slight decrease in grade 7, following by an increase in grade 8. Additionally, female students performed better than male students on all three abilities.

Then, a two-way ANOVA was conducted to compare the main effects of grade and gender, and the interaction effect between both variables on three abilities. The results showed that grade had main effect on all three abilities (F_analytical_ (4,1996) = 153.61, *p* < 0.001, η2 = .24; F_creative_(4,1996) = 30.05, *p* < 0.001, η2 = .06; F_practical_(4,1996) = 69.40, *p* < 0.01, η2 = .12) and gender only had a main effect on analytical (F(1,1996) = 10.76, *p* < 0.001, η2 = .01) and creative abilities (F(1,1996) = 15.89, *p* < 0.001, η2 = .01), but not on practical ability (F(1,1996) = .002, *p* = .965, η2 = .000). Post hoc comparisons were conducted to examine the differences between each grade, and the results showed that there were significant differences among the five grades on analytical ability. Whilst for creative ability and practical ability, the differences among five grades were also significant, except between grades 4 and 5, Additionally, there was no significant interaction between grade and gender.

Further, the latent mean score comparisons of analytical, creative, and practical ability were also reported in Table 9. Grade 4 and male students were chosen as reference groups, and their latent means were set to . The results suggested that grade and gender difference patterns reflected by latent mean scores were same to that observed means. 

## 4. Discussion

This study provides evidence of the reliability and validity of the Aurora Battery in China, as well as analyzes the performance of Chinese students on all three abilities of successful intelligence. 

### 4.1. The Validity and Reliability of Aurory-a Battery in China

The Aurora Battery is a corresponding test of successful intelligence, which comprises analytical, creative, and practical subtests, spanning images, words, and numbers domain (Chart et al. 2008). Researchers in multiple countries around the world have been using the Aurora test to assess cognitive abilities in students, with scholars in countries such as Great Britain and Saudi Arabia applying the test in the selection of gifted children (Mourgues et al. 2016; Tan et al. 2014). Hein and colleagues used the Aurora Battery to study the family environment and school environment of gifted children (Hein et al. 2014, 2015). Therefore, translating and revising the Chinese version of Aurora Battery can provide educators in China with a new method to assess students’ cognitive abilities from a broader perspective, and can better address students’ strengths and weaknesses. 

Confirmatory factor analysis indicated that a second-order factor model with a general factor explaining variation in cognitive abilities (i.e., analytical, practical, and creative thinking) was the most parsimonious model and yielded the best fit to the data. The structure is similar to the conclusion made by Iranian researchers (Aghababaei et al. 2016). They selected 400 gifted children in Iran and found that three specific ability factors can be extracted from Aurora subtests. A study by Aljughaiman and Ayoub (2012) in Saudi Arabia, explored the structure of Aurora Battery comprising all of the 17 subtests, suggesting a good fit for the three-ability model as well, but the specific domains within each subtest were not modeled as a single latent factor (Aljughaiman and Ayoub 2012). Mourgues and colleagues (2016) in the UK only adopted the creative subtest (including Figurative, Number Talk, Conversations, Multiple Uses, and Book Covers tests) of Aurora for research, and their study documented that the five creative subtests shared general skills of creativity, but domain-specific (Images, Numbers and Words) creativity did not describe the covariation among Aurora’s creative subtests (Mourgues et al. 2016). These findings are in line with Baer’s studies (Baer 1996, 2010), which have shown in some similar domain tasks, there still exists the possibility that individual’s performance is not uniform.

An alternative explanation could be both the types of testing items and the way to rate answers might influence whether successful intelligence appears to be more domain-specific. For instance, the Number Talk subtest is rated basing on students’ understanding of mathematical concepts reflected in the response, but students’ writing skills might also affect the quality of their responses, and consequently then influence the score. Another reason that successful intelligence does not appear to be more of domain-specific is related to the sample age. Some literature indicates that the disparities on domain-specific tasks and domain-general tasks occur as children get older, to be more specific, as individuals grow up, their talents might appear to be more domain-specific (Gathercole et al. 2004). In an eight-year longitudinal study, David and his colleagues (2017) discovered that individual’s performance among different intelligence domain are moderately stable when children are young (Geary et al. 2017). In our research, students were approximately around 12.3 years old, an age that may not have too much chance for them to show their talents within specific domain. Meanwhile, at the stage of primary and secondary schools, China currently emphasizes the holistic development of students, requiring the integrative development of moral, intellectual, physical, aesthetic, and labor development, and the development of students’ abilities in all aspects should be relatively balanced, so based on the educational condition in China, there was no domain differences. 

After identifying the best fit model, we furthered the analysis of the chosen model for MI tests. Among the tested models, the first-order scalar model across gender was not fully supported, so a partial scalar invariance model was further examined, and the results showed that by allowing the variation of intercepts on Metaphor and Toy Shadow subtest, the changes in CFI turned out to be insignificant. Consequently, our results confirmed MI of the Aurora Battery across grades and gender, implying that the constructs of Aurora Battery work similarly in male and female groups, as well as students from different grades. Males and females, and students at different grades, can all understand the items of Aurora Battery effectively, and the obvious disparities in Aurora scores across grades and gender can be attributed to their performance of successful intelligence, not because they are males or females, or the influence of their grades. 

Aurora Battery and its subscales all showed to have significant, medium to strong correlations with the TONI-2 Intelligence Test, the EPoC creativity test and academic scores. The Aurora Battery aims to measure the individuals’ ability to achieve their goals in real life and it has three integrated key features, which emphasis on the ability of analyzing, decision-making and problem-solving. The evidence strongly supported the validity of Aurora battery, and it can be adopted in China as an effective approach to recognize students’ strength and weakness from diverse aspects, which accords with the kernel of China’s educational assessment reform. 

The scale reliability analysis showed that the Cronbach’s alpha and McDonald’s omega value of the total composite score (i.e., across items of all fifteen subtests) was .84 and .87, respectively, indicating the scale’s internal reliability with acceptable to good consistency.

### 4.2. The Descriptive Results of Successful Intelligence in Chinese Students

Since measurement invariance across grades and gender were confirmed, we then explored students’ developmental trends and gender differences on successful intelligence reflected by the Aurora Battery. Through analysis, we discovered that students’ analytical and practical abilities increase gradually across grades, this is because analytical and practical ability are inseparable from an individual’s knowledge and intelligence levels (Shi 2012), and it is usually found to increase over time (Cianciolo et al. 2006; Flynn 2007; Gubbels 2016). 

However, for creativity, the result presented an upward trend from grades 4 to 6, whereas with a slight decrease in grade 7, following by an increase through grade 8. These finding was similar to some other research (Claxton et al. 2005; Smith and Carlsson 1990; Richards 1991; Smolucha and Smolucha 1985). Students’ creativity slumps during middle childhood can be explained by the rising pressure of study, fierce academic competition, and the results of socialization and teaching of conformity (Camp 1994; Saggar et al. 2019). When entering grade 7, students have to study within a new learning environment and face with an increasing learning burden (Du et al. 2014), and in order to prepare for future enrollment in higher education, they need to more focus on the accuracy of their work instead of the aesthetic appeal, so that their creativity might be impaired (Rosenblatt and Winner 1988; Saggar et al. 2019). The development of creativity is also related to students’ ability to control tasks; studies have shown that students can spend more time in extracurricular reading and problem-exploring when they can quickly complete their learning tasks, thus seizing more opportunities to be creative (Liu et al. 2015). At grade 7, students are experiencing a transition from primary school to middle school, where the learning methods and learning difficulties are totally different, so students need more time to adapt and complete the difficult learning tasks. As a result, they often cannot have enough time for extracurricular reading and interest exploration activities. Therefore, students’ creativity may have a decrease at grade 7. After one-year’s adaptation to the study in middle school, students can better arrange their learning tasks and extra-curricular activities, which will be beneficial for their creativity development and thus lead to the resurgence of their creative task performance in grade 8.

Meanwhile, this research discovers that female students outperformed male students significantly across analytical abilities. Analytical ability is more of the academic performance, and numerous studies have confirmed that girls always obtain higher scores than their counterparts (Clifford 2018; Duckworth and Seligman 2006). This might be attributed to their brain differences, specifically, girls generally have stronger neural connectors in their temporal lobes than boys, and the stronger connectors will be conducive to sensorially detailed memory storage and class listening (Clifford 2018). Consequently, at the early stage of schooling, when courses are not very difficult, girls usually perform better. Another explanation might be that girls are more self-disciplined and treat study more carefully (Duckworth and Seligman 2006), which will result in better academic performance. 

As for gender differences on creativity, we discovered that girls scored significantly higher than boys in terms of creativity. This is quite similar to Chung’s study, which suggested that girls have higher scores in verbal tasks and graphic tasks than boys (Cheung and Lau 2010) and Aurora’s creativity test puts more focus on verbal fields. Girls have more advantages than boys when it comes to verbal expression, so they will have higher self-concept and self-efficacy (Liu 2004). 

The result also suggested that there is no gender difference on practical ability, which is consistent with that of Somech and Bogler (1999). A possible explanation is that boys and girls are equipped with distinct advantages on practical tasks. Boys tend to be more open, flexible, and good at operation, while girls may be more sociable, excellent in dealing with peer relationships, and good at listening to others’ opinions. Therefore, boys and girls have their own advantages on practical ability.

## 5. Conclusions

In conclusion, among the tested models, a second-order factor model in which the three ability factors serve as indicators of a general factor provided an acceptable fit to the data. It demonstrated that the Aurora Battery is an effective assessment tool for identifying high scoring in analytical ability, practical skill, and creative thinking, which is correspond to Sternberg’s theory of successful intelligence. The scale reliability analysis showed that the Cronbach’s alpha of the whole test was .84, indicating the scale’s internal reliability with good internal consistency. The criterion-related validity analysis showed that the battery and its three subscales had good criterion validity. The descriptive result of successful intelligence in Chinese students indicated that there were gender differences and grade differences on analytical ability, practical skill, and creative thinking abilities.

There were also several limitations in our study. One limitation of this study was the sample size, as China is a multi-ethnic country, so in future research, students from diverse ethnic groups should also be incorporated. In addition, the test-retest reliability of Aurora Battery was not examined. Adequate test-retest reliability can ensure the stable construct of the test, and this will be useful for repeated-measures or time-series study designs, so further studies can collect data at different time points to tackle this issue. Moreover, though TONY-2, EPoC, and academic scores demonstrated the criterion-related validity of Aurora, further measures with various domains and other abilities should be supplemented. For example, the WISC test, which includes similar domains (words, images and numbers domain) with Aurora Battery can be adopted to examine the validity of Aurora Battery, and tasks that can reflect the individual’s real-word practical abilities should be adopted. Besides, students were not provided with equal time to finish the subtests of Aurora Battery, which might influence the accuracy among comparisons on analytical and practical ability between different grades. Furthermore, as explanations for grade and gender differences were deduced from theoretical perspective, further investigations could be conducted, to ascertain whether the mentioned reasons truly influence students’ successful intelligence.

## Figures and Tables

**Table 1:** Basic sample demographics (N = 2007).

Characteristic	Indicators	*n*	Percentage
Area	East	1008	50.2
	Center	573	28.6
	West	426	21.2
Gender	Boy	1125	56.1
	Girl	881	43.9
Grade	4th	334	16.6
	5th	425	21.2
	6th	422	21.0
	7th	464	23.1
	8th	362	18.0

**Table 2:** The Chinese version of Aurora Battery.

	Analytical	Creative	Practice
Images	Floating Boats (10 items) (MC)	Book Covers (5 items) (OE)	Paper Cutting (10 items) (MC)
		Multiple Uses (5 items) (OE)	Toy Shadows (8 items) (MC)
Words	Words That Sound the Same (Homophones) (16 items) (RW)	Conversations (10 items) (OE)	Decisions (3 items) (RW)
	Metaphors (9 items) (OE)	Figurative Language (10 items) (MC)	
Numbers	Story Problems (Algebra) (7 items) (RW)	Number Talk (7 items) (OE)	Maps (10 items) (RW)
	Number Cards (Letter Math) (5 items) (RW)		Money Exchange (5 items) (RW)

Notes. MC: Multiple Choice. OE: Open-ended items that need to be scored by an individual using a rating scale and scoring rubric. RW: Answers are either Right or Wrong.

**Table 3:** Fit statistics for alternative factor models of the Aurora Battery (N = 2007).

	χ^2^ (df)	CFI	TLI	RMSEA	SRMR
Model 1	970.25(87) ***	.879	.854	.071	.056
Model 2	670.68(87) ***	.920	.903	.058	.051
Model 3	537.392(76) ***	.937	.913	.055	.042

Notes. χ^2^ = Chi-square, df = degree of freedom, CFI = Comparative Fit Index, TLI Tucker-Lewis index, RMSEA = Root Mean Square Error of Approximation, SRMR = Standardized Root Mean Square Residual, *** p < .001.

**Table 4:** Decomposition of Variance for model 2 (N = 2007).

Subtests	Aurora Abilities					
	Analytical		Creative		Practical	
	λ	*var*	λ	*var*	λ	*var*
Algebra	.69	.48				
Floating Boats	.65	.42				
Metaphors	.48	.23				
Letter Math	.52	.27				
Homophones	.69	.48				
Paper Cutting					.60	.36
Decisions					.56	.31
Maps					.56	.31
Money					.74	.55
Toy Shadows					.48	.23
Figurative			.45	.20		
Conversations			.56	.31		
Number Talk			.43	.18		
Multiple Uses			.63	.40		
Book Covers			.54	.29		

Notes. λ = standardized factor loading. var = partial subtest variance explained by the latent factor. All standardized factor loadings were significantly different from zero at *p* < 0.001.

**Table 5:** Measurement invariance across grades.

Models	χ^2^ (df)	CFI	RMSEA	90% CI	Comparison	ΔCFI	ΔRMSEA
M1: Configural invariance	1054.555(435)	.885	.06	.055–.064	-	-	-
M2: First-order metric	1137.003(483)	.878	.058	.054–.062	M2 vs. M1	−.007	−.002
M3: Second-order metric	1109.873(491)	.885	.056	.052–.060	M3 vs. M2	.008	−.002
M4: First-order scalar	1121.644(539)	.891	.052	.048–.056	M4 vs. M3	.006	−.004
M5: Second-order scalar	1126.369(549)	.892	.051	.047–.055	M5 vs. M4	.001	−.001
M6: Residual (obs)	1150.909(576)	.893	.050	.046–.054	M6 vs. M5	.001	−.001
M7: Residual (lat)	1152.187(588)	.895	.049	.045–.053	M7 vs. M6	.002	.001

Notes: CFI Comparative fit index; RMSEA Root mean square error of approximation; 90% CI 90%. Confidence interval of the RMSEA.

**Table 6:** Measurement invariance across gender.

Models	χ^2^ (df)	CFI	RMSEA	90% CI	Comparison	ΔCFI	ΔRMSEA
M1: Configural invariance	639.466(174)	.909	.052	.047–.056	-	-	-
M2: First-order metric	658.395(186)	.907	.05	.046–.055	M2 vs. M1	−.002	−.002
M3: Second-order metric	657.401(188)	.908	.05	.046–.054	M3 vs. M2	.001	0
M4 First-order scalar	784.23(200)	.885	.054	.050–.058	M4 vs. M3	−.023	.004
M4a: First-order partial scalar	699.081(190)	.900	.052	.048–.056	M4a vs. M3	−.008	.002
M5: Second-order partial scalar	708.169(192)	.899	.052	.048–.056	M5 vs. M4a	−.001	0
M6: Residual (obs.)	727.249(207)	.898	.050	.046–.054	M6 vs. M5	−.001	−.002
M7 Residual (lat.)	738.420(210)	.896	.050	.046–.054	M7 vs. M6	−.002	0

Notes: CFI Comparative fit index; RMSEA Root mean square error of approximation; 90% CI 90%. Confidence interval of the RMSEA.

**Table 7:** Correlation matrix among Aurora, TONY-2 and EPoC (N = 443).

	1	2	3	4	5
1 Analytical ability	-				
2 Practical ability	.62 ***	-			
3 Creative ability	.68 ***	.51 ***	-		
4 Aurora score	.95 ***	.77 ***	.83 ***	-	
5 EPoC	.48 ***	.33 ***	.53 ***	.53 ***	-
6 TONI-2	.48 ***	.45 ***	.42 ***	.52 ***	.36 ***

Notes. *** *p* < 0.001.

**Table 8:** Correlation matrix among Aurora, and academic scores (N = 188).

	5	6	7
1 Analytical ability	.62 ***	.48 ***	.64 ***
2 Practical ability	.53 ***	.50 ***	.61 ***
3 Creative ability	.45 ***	.40 ***	.50 **
4 Aurora score	.65 ***	.57 ***	.72 ***
5 Chinese	-	.42 ***	.80 ***
6 Math		-	.42 ***
7 Academic score			-

Notes. *** *p* < 0.001.

**Table 9:** Descriptive statistics of Aurora abilities (N = 2007).

		Analytical			Creative			Practical		
		*M*	*SD*	Latent Mean Score Difference	*M*	*SD*	Latent Mean Score Difference	*M*	*SD*	Latent Mean Score Difference
Grade	4th	45.49	5.95	0	47.88	5.35	0	46.77	6.46	0
	5th	47.07	6.07	.397 ***	48.36	6.16	.023	47.48	6.67	.195
	6th	49.29	6.26	.884 ***	50.95	5.44	.618 ***	50.40	6.34	.754 ***
	7th	52.43	6.49	1.622 ***	49.87	6.95	.390 ***	51.28	6.58	.939 ***
	8th	55.21	5.67	2.260 ***	52.41	7.64	.865 ***	53.64	6.65	1.396 ***
Gender	male	49.42	6.88	0	49.33	6.78	0	49.85	6.72	0
	female	50.70	7.08	.186 ***	50.63	6.24	.225 ***	50.13	7.26	.081

Notes. Analytical score was averaged by *T*-scores of Story Problems, Floating Boats, Metaphors, Number Cards (Letter Math) and Words That Sound the Same (Homophones). Creative score was averaged by *T-*scores of Interesting (Figurative) Language, Conversations, Multiple Uses, Book Covers and Number Talk. Practical score was averaged by *T*-scores of Paper Cutting, Decisions, Maps, Money Exchange and Toy Shadows. *** p < 0.00.

## Data Availability

The data presented in this study are available on request from the corresponding author. The data are not publicly available due to participant privacy.
